# Characterization of the Role of eIF4G in Stimulating Cap- and IRES-Dependent Translation in *Aplysia* Neurons

**DOI:** 10.1371/journal.pone.0074085

**Published:** 2013-09-03

**Authors:** John Dyer, Wayne S. Sossin

**Affiliations:** Department of Neurology and Neurosurgery, Montreal Neurological Institute, McGill University, Montreal, Quebec, Canada; Florida State University, United States of America

## Abstract

The rate-limiting step(s) of translation in the nervous system have not been clearly identified. We have been examining this question in the cell body of the *Aplysia* sensory neuron, where translational regulation is important for the regulation of synaptic strength. In the present study, we examined the role of the adaptor protein eIF4G. We cloned *Aplysia* eIF4G (Ap4G) and Ap4G contains all the standard metazoan eIF4G protein–protein interaction domains. Overexpressing Ap4G in *Aplysia* sensory neurons caused an increase in both cap-dependent and internal ribosome entry site (IRES)-dependent translation using a previously characterized bicistronic fluorescent reporter. Unexpectedly, measurement of overall translation using the methionine analog, L-azidohomoalanine, revealed that overexpression of Ap4G did not lead to an increase in overall translation rates. Indeed, the effect of Ap4G on the bicistronic reporter depended on the presence of an upstream open reading frame (uORF) in the 5’ UTR encoded by the vector. We have previously shown that Mnk strongly decreased cap-dependent translation and this depended on a putative 4G binding domain. Here we extend these results showing that even in the absence of the uORF, overexpression of Mnk strongly decreases cap-dependent translation and this depends on the Mnk binding site in eIF4G. Similarly, an increase in cap-dependent translation seen with overexpression of elongation factor 2 kinase did not depend on the uORF. Overall, we show that eIF4G is rate limiting for translation of an mRNA encoding an uORF, but is not generally a rate-limiting step for translation.

## Introduction

In many cases, translational control is studied in the context of cell size and cell proliferation, since in most cell lines and in cancerous cells, translational control is critical in determining whether or not the cell decides to double its proteome and divide [1,2]. Translational control is also critical in times of stress, when most translation is reduced, but critical stress responsive proteins are now translated [3]. In contrast, translation in mature neurons is mainly controlled by external signals altering neuronal properties by changing the proteome, and thus is important for regulating synaptic plasticity [4,5].

We have been studying translational control using the model system of the *Aplysia* *californica* sensory neuron. In particular, we have been interested in how extracellular signals alter translational control factors to change the proteome of the neuron during synaptic plasticity. In many of these studies, we have been using a bicistronic reporter with enhanced cyan fluorescent protein (eCFP) being driven by cap-dependent translation and enhanced yellow fluorescent protein (eYFP) driven by a verified internal ribosome entry site (IRES) derived from the *Aplysia* egg-laying hormone mRNA [6]. However, during these studies it has become clear that more fundamental questions about the regulation of translation in neurons remain open. For example, while eIF4E is thought to be a rate-limiting factor in many cases, overexpression of eIF4E did not increase cap-dependent translation in *Aplysia* sensory neurons [7]. In contrast, overexpression of the eIF4E kinase, Mnk, led to a strong decrease in cap-dependent translation that depended on eIF4G binding [8]. eIF4G has been reported to be important for both cap-dependent and IRES-dependent translation [9,10]. In the present study, we examined the role of eIF4G in translation of cap and IRES-dependent translation in the *Aplysia* sensory neuron. Surprisingly, we found that eIF4G was rate-limiting for cap-dependent translation only in the context of an upstream open reading frame (uORF). Given the large number of mRNAs encoding uORFs, this suggests a novel mechanism for translational regulation in neurons. 

## Methods

### Animals

*Aplysia* *californica* (70-150 g) were obtained from the University of Miami National Institute of Health *Aplysia* Resource Facility (Miami, FL) and maintained in an aquarium for at least 2 days before experimentation. Prior to dissection, animals were placed in a bath of isotonic MgCl_2_-artificial seawater (1:1, vol/vol) and then anesthetized by injection with isotonic MgCl_2_ solution. Ganglia were isolated from the animal and placed in L15 (Sigma) before use.

### Sensory Neuron Cultures and Expression Plasmid Injection

Sensory neurons from the pleural sensory neuron clusters of 1 or 2 *Aplysia* were isolated, plated and injected with plasmid DNA as described in (Farah and Sossin, 2011). In each experiment, cells from each sensory cluster were distributed across each treatment group. The neurons were incubated in L15 and hemolymph (25%) at 19 C for 16 to 24 hours prior to and for 12 to 48 hours after injection.

### *Aplysia* eIF4G Cloning and Plasmid Construction

Using the eIF4G sequence of the invertebrate *Lottia* *gigantea* , several large *Aplysia* contig sequences with considerable homology were identified in the NCBI database. The entire sequence was amplified in seven overlapping segments by PCR from *Aplysia* nervous system cDNA (RNAqueous, Ambion; SuperScript II, Invitrogen) or by 5’ RACE (FirstChoice RLM-RACE, Ambion), except for one short region (nuc 2524 to 2573 from initiating ATG) which we were unable to amplify. The genomic sequence for this region was incorporated into the reverse primer used to amplify the adjacent upstream segment; there are no apparent exon/intron boundaries in this region, but it consists almost entirely of G or C residues. The *Aplysia* eIF4G (Ap4G) segments were cloned into pJET (Fermentas), joined into two large segments using overlapping PCR then transferred sequentially into the *Aplysia* expression vector, PNEX3 [11] using unique restriction sites. Three key residues in the eIF4E binding domain of Ap4G were mutated (Y808A, L813A, M814A) in pNEX3-Ap4G by site-directed mutagenesis (Phusion polymerase, NEB; DPN I, NEB) to produce pNEX3-Ap4GΔ4E. The C-terminal of Ap4G minus almost all of the Mnk-binding domain ( *Aplysia* 1583-1767 aa) was synthesized by overlapping PCR and inserted into pNEX3-Ap4G between NotI and KpnI sites to produce pNEX3- eIF4G∆Mnk. The ten C-terminal amino acids of Ap4G were retained to maintain antigenicity towards the Ap4G C-terminal antibody (see below). The modified bicistronic expression vector (pNEX3-ΔuORF-C _IRES_Y) was made by digesting our original bicistronic expression vector (pNEX3-C _IRES_Y) with SphI then blunting with Klenow and religating.

### Translation Rate Measurement

Pleural sensory cells were injected with one of the two fluorescent bicistronic pNEX3 expression vectors (100 ng/µl in 0.25% Fast Green aqueous) to measure both cap-dependent translation (eCFP expression) and IRES-dependent expression (eYFP expression) from the IRES in *Aplysia* egg-laying hormone [6]. The effects on these translation rates of overexpressing Ap4G or its mutants, with or without overexpressing Ap4E [6] or ApMnk [8], were determined by coinjecting the pNEX3 expresssion vectors for these proteins and one of the bicistronic reporters (each at 100 ng/µl, except pNEX3-Mnk at 10 ng/µl). Empty pNEX3 vector was injected in control groups so that the concentration of DNA injected was similar.

In some experiments, the overall cellular translation rate was also measured by the incorporation of the methionine analogue, L-azidohomoalanine (AHA) (Invitrogen) [12]. This method was adapted for use in *Aplysia* . At 12, 24 or 48 hours after pNEX3 injections, the methionine concentration of the culture media was reduced to approximately 50 μM by reducing the culture media to approximately 100 μl (volume of the culture dish’s well containing the neurons) before adding 2 ml of a methionine-free media (equal levels of other amino acids; no hemolymph). Cells were incubated at 19^0^C for 120 minutes before reducing the media volume to 100 μl and adding 100 μl of 100 μM AHA in methionine-free media. Cells were incubated at 19^0^C for 90 minutes during which the bicistronic vector fluorescence was measured. After 45 minutes, 2 ml of isotonic buffer was added, removed and the cells fixed, permeabilized and quenched as described for ICC. The incorporated AHA was detected by conjugation to alkyne-Alexa Fluor 555 (0.2 μg/μl final concentration) (Click-IT, Invitrogen) and imaging under the fluorescent microscope. In each experiment, nontranslational AHA incorporation was determined in a separate dish of neurons incubated with 250 μM emetine for 15 minutes before and during the AHA incubation. The mean AHA incorporation in the presence of emetine was subtracted from the AHA incorporation determined in each neuron.

### Antibodies

The C-terminal peptide sequence of *Aplysia* eIF4G (QLTQFFTWLSENEEPEAAS-COOH) was used to generate an antibody in rabbits. Antibodies for detecting the *Aplysia* homolog of eukaryotic initiation factor 4E were previously characterized [13] Rhodamine-Red or Alexa-Fluor-647 conjugated goat anti-rabbit IgG (Invitrogen) were used as secondary antibodies (1:1000).

### Immunocytochemistry

Sensory neurons were fixed (4% PFA, 4% sucrose in PBS, pH 7.4) for overnight at 19C, permeabilized (30% sucrose, 0.1% Triton X-100 in PBS) for 10 minutes and quenched (50mM NH_4_Cl in PBS) for 30 minutes. The cells were blocked (10% Normal Goat Serum (Sigma), 0.5% Triton X-100 in PBS) for 30 minutes, incubated overnight at room temperature with the primary antibody (1:500 in block for eIF4G and 1:750 for eIF4E), washed four times with PBS, incubated with the secondary antibody in block for one hour, washed four times with PBS, and imaged under the fluorescent microscope.

### Imaging and Quantitation

Cells were imaged using a Zeiss Axio Observer Z1 microscope (Plan Apochromat 40X/0.95 objective) equipped with Zeiss 47 (cyan), Zeiss 46 (yellow), Zeiss 20 (red) and Zeiss 50 (far-red) filters. In each neuron for each fluorophore, the maximum fluorescence intensity was measured by the ImageJ (NIH) software program. The mean maximum fluorescence intensity of each fluorphore in non-injected neurons from the same experiment was subtracted to remove background cell fluorescence. An experiment is defined as using the same group of sensory neurons (neurons from the same batch of animals) that are injected and imaged/stained under the same conditions. For each experiment the values for all neurons were normalized to the average value of the control neurons for that experiment and this allows us to compare these normalized results across different experiments. For each result, both the n (number of neurons) and the number of independent experiments that these neurons were derived from are presented. Each result comes from at least three independent experiments using different batches of sensory neurons. For experiments with two groups, Two-tailed Student’s *t*-tests with Welch’s and Bonferroni corrections (for unequal variances between groups and multiple tests, respectively) were used to determine statistical significance. For experiments with more than two groups, a Kruskal Willis non-parametric ANOVA was performed with post-hoc Dunn tests to compare between different groups using Instat (GraphPad Software). Data from some experiments appear in more than one figure and these instances are detailed in the figure legends.

## Results

### Cloning of Aplysia eIF4G

**Figure 1:**
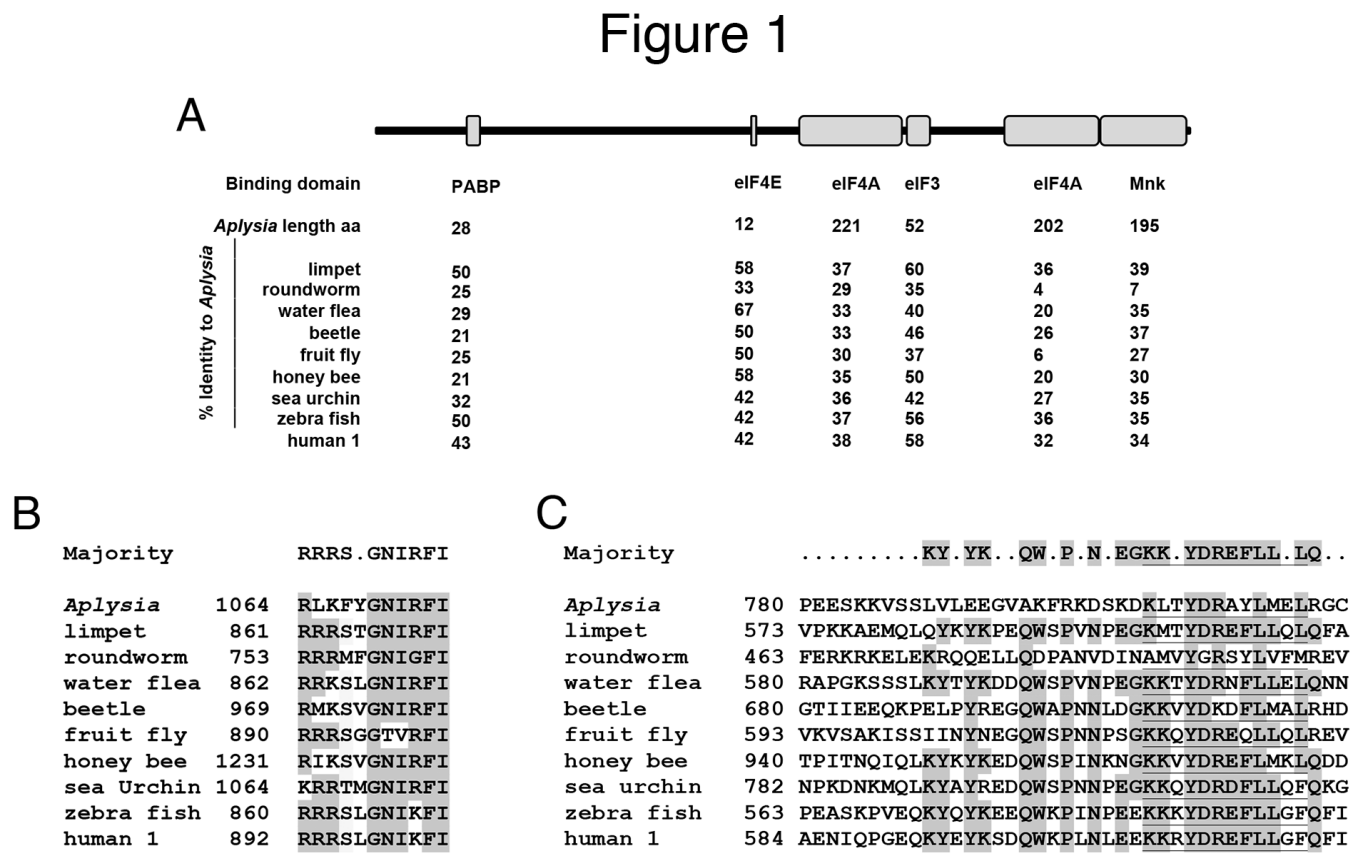
Characteristics of *Aplysia* eIF4G. The major protein binding domains of *Aplysia* eIF4G (Ap4G) are well conserved. Diagram of eIF4G shows the placement and relative sizes of the domains known to bind other proteins involved in translation: polyA binding protein (PABP) (*Aplysia* 205-232), eIF4E(*Aplysia* 805-816), eIF4A-1(*Aplysia* 899-1119), eIF3(*Aplysia* 1130-1182), eIF4A-2(*Aplysia* 1383-1584), Mnk (*Aplysia* 1583-1767); the numbering is relative to the first possible initiating Met in *Aplysia* sequence and *Aplysia* amino acid ranges are based on alignment with eIF4G orthologues using ClustalW software (LaserGene). The number of amino acids in each domain in Ap4G is given underneath. The percentage of identical amino acids in these binding domains from an evolutionarily disparate group of species is shown: owl limpet (*Lottia gigantean*, jgiLotgi234084), pig roundworm (*Ascaris suum*, GQ373389), water flea (*Daphnia pulex*, EFX67152), red flour beetle (*Tribolium castaneum*, EFA03765), fruit fly (*Drosophila melanogaster*, NP_524640), western honey bee (*Apis mellifera*, NP_001177977), common purple sea urchin (*Sphaerechinus granularis*, CAG23924), zebra fish (*Danio rerio*, NP_001073669) and human 4G-1 (NP_886553). **B**. Region surrounding PAK2 phosphorylation site and **C**. homology in region of eIF4E binding site conserved in all species examined except for *Aplysia* and roundworm. eIF4E binding site is underlined. Amino acids identical to majority (>5) are shaded.

*Aplysia* eIF4G (Ap4G) was cloned by a combination of bioinformatics, PCR and 5’ RACE (See Methods). eIF4G is an adaptor that contains a number of identified protein–protein interaction domains and Ap4G contains all domains present in vertebrate eIF4G1 and eIF4G2, including those that bind poly-A-binding protein (PABP), eIF4E, eIF4A, eIF3 and Mnk (Figure 1A). We also screened the *Aplysia* sequence for conservation of identified and studied phosphorylation sites in vertebrate eIF4Gs. Two of the major sites that have been examined, Ser 1108, a serum-activated and rapamycin sensitive site [14], and Ser 1232, an ERK site [15] (numbering is for human eIF4G1), are not conserved in *Aplysia* or in most other invertebrate species (data not shown). One identified site that is highly conserved in most invertebrates, a p21-activated kinase 2 (PAK2) phosphorylation site [16], is also absent in *Aplysia* , despite its presence in the limpet, *Lottia*. Interestingly, PAK2 was found to bind to eIF4G near the eIF4E binding site and compete with eIF4E for binding to eIF4G [16] and *Aplysia* also lacks a conserved region on the N-terminal side of the consensus eIF4E binding site that is present in all species that contain the PAK2 phosphorylation site (Figure 1B). One site that may be conserved in *Aplysia* is the recently described Casein 2 kinase site, Ser 1239 [15].

We raised an antibody to the carboxy-terminal sequence of eIF4G and it recognized a protein of the expected molecular weight (195000 kD) in *Aplysia* ganglia (Figure S1). A number of additional bands were recognized, although it is unclear if these represent eIF4G degradation products or nonspecific bands.

### Overexpression of eIF4G increased both cap-dependent and cap-independent translation using a bicistronic reporter in *Aplysia* neurons

**Figure 2:**
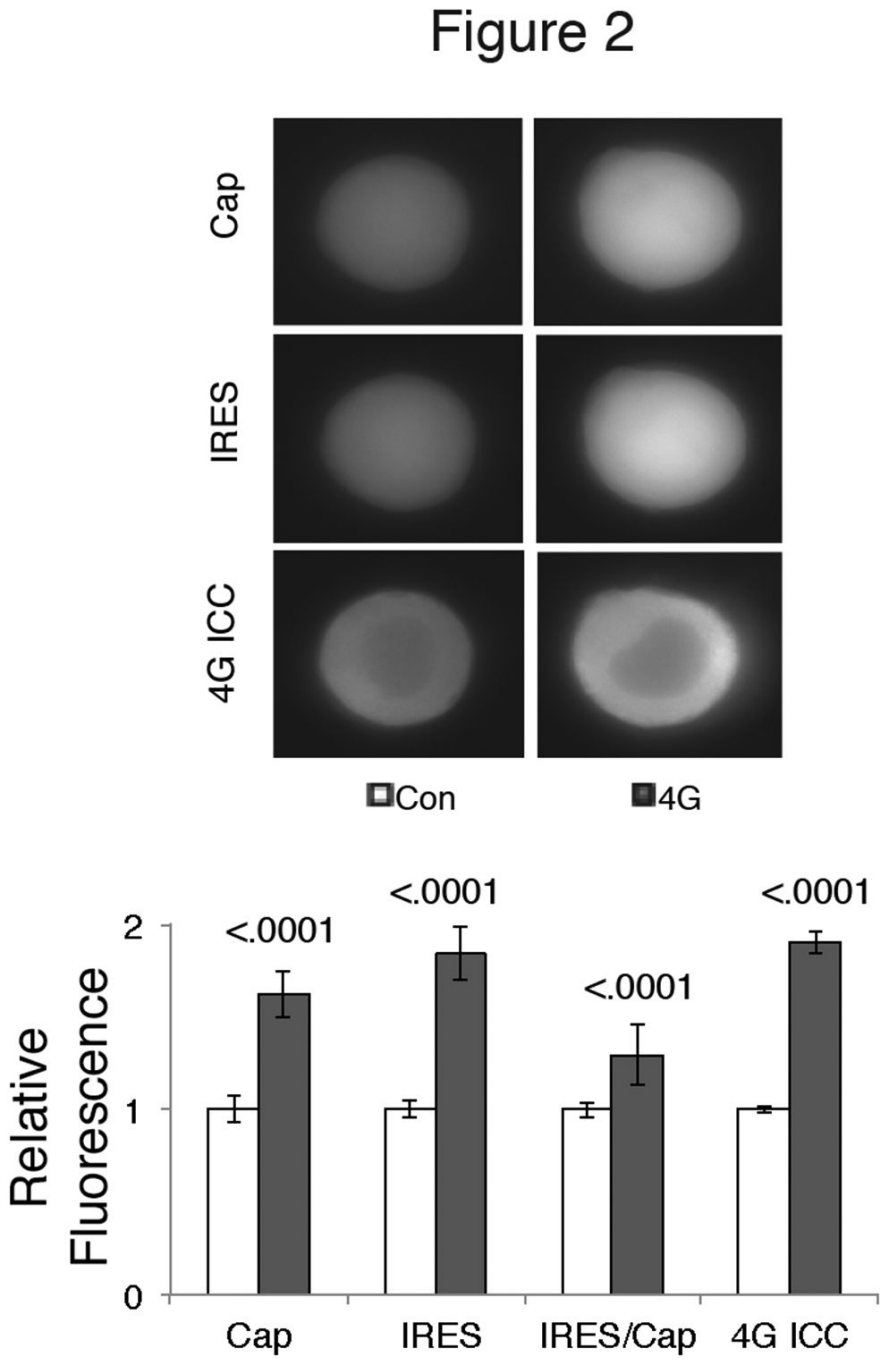
Overexpressing Ap4G in *Aplysia* neurons increases cap- and IRES-dependent translation from a reporter construct. Cultured *Aplysia* sensory neurons were co-injected with a bicistronic fluorescent reporter and either empty expression vector (Con) or Ap4G (4G). The photos are representative neurons, 48 hours later; the top row shows cyan fluorescence used as a reporter of cap-dependent translation (Cap), the middle row shows yellow fluorescence used as a reporter of IRES-dependent translation (IRES) and the bottom row shows red fluorescence from immunostaining for eIF4G (4G ICC) in the same neurons. The histogram of means (normalized to the respective mean of control cells) and SEMs shows the fluorescent protein expression from the groups of the representative neurons. The ratio of IRES- to cap-dependent translation was calculated (IRES/Cap). The values, SEM and Ns are: control (Cap 1.00 ± 0.07, IRES 1.00 ± 0.05, IRES/CAP 1.00 ± 0.04, 4G 1.00 ± 0.04, n=159 neurons from 16 experiments for CAP, IRES and IRES/CAP, n= 85 neurons from 10 experiments for 4G) eIF4G (Cap 1.62 ± 0.13, IRES 1.84 ± 0.14, IRES/CAP 1.29 ± 0.16, 4G 1.90 ± 0.06, n=180 neurons from 16 experiments for CAP, IRES and IRES/CAP, n=88 cells for 4G) Student’s *t* test p values (with Welch’s and Bonferroni’s corrections) between control and eIF4G expression are shown over the bars.

To determine if levels of eIF4G are rate-limiting for translation in *Aplysia* neurons, we overexpressed Ap4G in isolated *Aplysia* sensory neurons in conjunction with a bicistronic translational reporter (pNEX3-C _IRES_Y), in which eCFP is translated by cap-dependent translation, whereas eYFP is translated by cap-independent translation through a verified cellular IRES [6,8]. Unlike previous results with eIF4G’s partner, eIF4E [7], over-expression of eIF4G significantly increased cap-dependent translation in this construct (Figure 2). eIF4G also increased IRES-dependent translation from the bicistronic construct (Figure 2). Overall, the IRES/Cap ratio was increased suggesting a slightly more important effect of eIF4G overexpression on IRES-mediated translation. Overexpression of eIF4G was confirmed with the antibody raised to *Aplysia* eIF4G (Figure 2).

**Figure 3:**
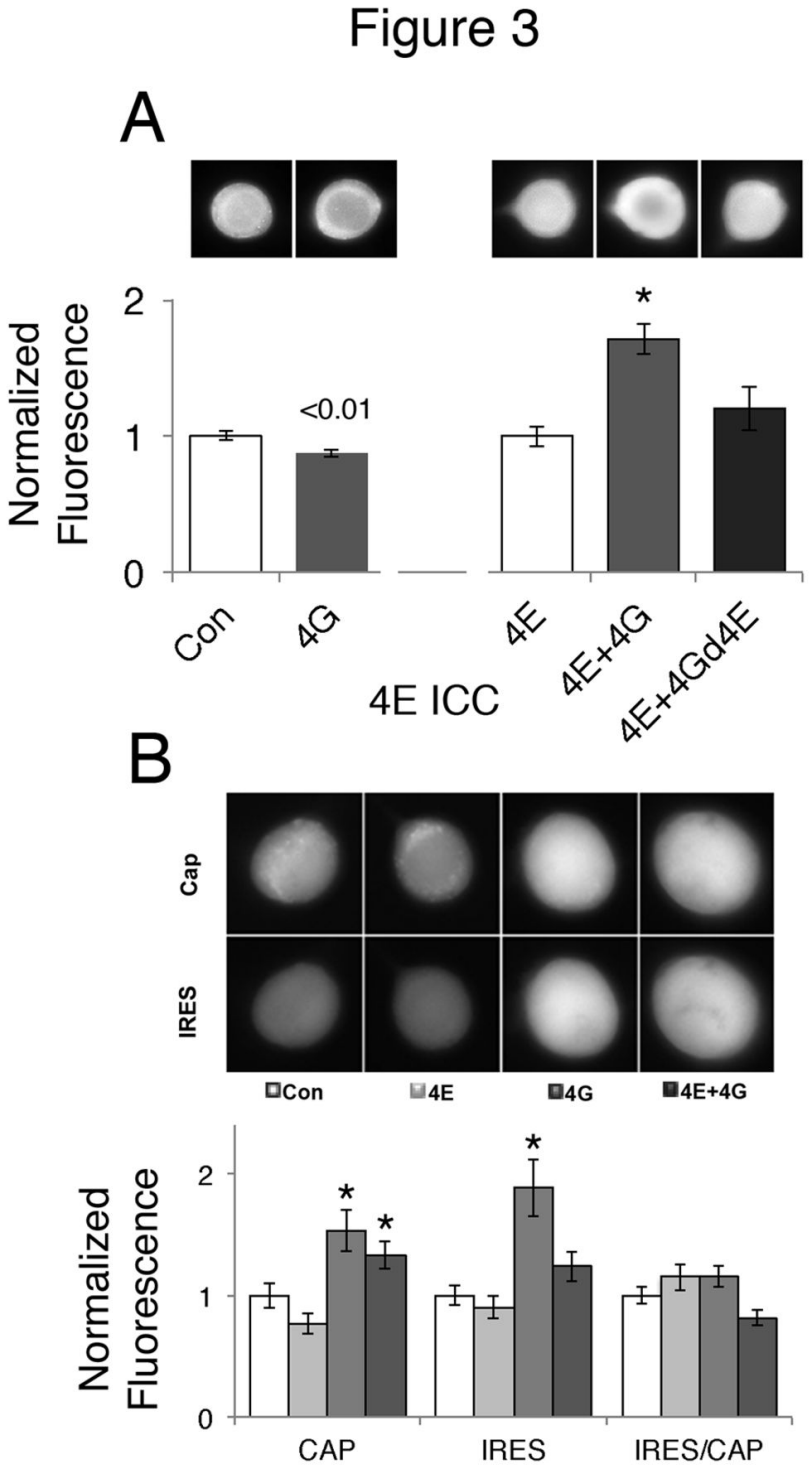
Overexpressing Ap4G stabilizes overexpressed Ap4E, but Ap4E does not contribute to increase in cap-dependent translation seen with Ap4G. **A**) Cultured *Aplysia* sensory neurons were injected with either empty expression vector (Con), Ap4G (4G), Ap4E (4E), Ap4E plus Ap4G (4E+4G) or Ap4E plus Ap4G with mutated eIF4E-binding site (4E+4GΔ4E). Representative neurons show red fluorescence from immunostaining for eIF4E, 48 hours later. Histogram is means (4G normalized to Con; 4E+4G and 4E+4GΔ4E normalized to 4E) and SEMs of the groups of the representative neurons. The values, SEM and Ns are: endogenous 4E, (control 1.00 ± 0.03, n=49 neurons from three experiments; 4G 0.87 ± 0.03, n=51 cells from three experiments) overexpressed 4E (4E 1.00 ±0.08, n=33 neurons from three experiments, 4E+4G 1.71 ± 0.12, n=36 neurons from three experiments, 4E + 4Gd4E 1.20 ± 0.16, n=42 neurons from three experiments. The experiments with endogenous 4E were from a subset of the experiments displayed in Figure 2. Student’s *t* test p values (with Welch’s and Bonferroni’s corrections) are shown over the bars for the comparison between control and 4G. For the comparison between control (4E), 4E + 4G, and 4E + 4Gd4E a non-parametric Kruskal-Wallis ANOVA was performed (KW statistic =22.6, p<0.001]. Dunn’s post Hoc tests showed that 4E + 4G is different from all other groups (both p<0.01, * in figure) but 4E is not significantly different from 4E + 4Gd4E (p >0.05). **B**) Cultured *Aplysia* sensory neurons were co-injected with a bicistronic fluorescent reporter and either empty expression vector (Con), Ap4E (4E), Ap4G (4G) or Ap4E plus Ap4G (4E+4G). Representative neurons (48 hours later) show cyan fluorescence (cap-dependent translation, Cap) and yellow fluorescence (IRES-dependent translation, IRES) in the same neurons. Histogram is means (normalized to Con) and SEMs from the groups of the representative cells. The ratio of IRES- to cap-dependent translation was calculated (IRES/Cap). The values, SEM and Ns are: Con (Cap 1.00 ± 0.10, IRES 1.00 ± 0.08, IRES/CAP 1.00 ± 0.07, n=54 cells from 4 experiments_; 4E (Cap 0.77 ± 0.09, IRES 0.90 ± 0.09, IRES/CAP 1.15 ± 0.11, n=56 cells from four experiments); 4G (Cap 1.53 ± 0.17, IRES 1.89 ± 0.23, IRES/CAP 1.16 ± 0.09, n=66 cells from four experiments); 4E + 4G (Cap 1.33 ± 0.11; IRES 1.24 ± 0.12; IRES/CAP 0.82 ± 0.07, n=53 cells from four experiments. These experiments represent a subset of the experiments used to calculate the effect of eIF4G vs Control in Figure 2. For the comparison between groups a non-parametric Kruskal-Wallis ANOVA was performed for CAP, IRES and the IRES/CAP ratio and then Dunn’s post-Hoc tests were performed to see if groups were different than control: Cap (KW statistic = 26.4, p<0.001. Both 4G and 4E + 4G were different than control, *, p<0.05); IRES (KW statistic = 16.6, p<0.001, Only 4G was significantly different than control *, p<0.05); CAP/IRES (KW statistic =8.84, p>0.05).

One possible mechanism to explain the increase in cap-dependent translation by overexpression of eIF4G would be a concomitant increase in eIF4E levels. We previously found that it was difficult to increase levels of eIF4E without co-expression of an eIF4E -binding partner, and that over-expression of eIF4E-binding protein (4EBP) increased endogenous levels of eIF4E [7]. However, over-expression of eIF4G actually led to a small, but significant decrease in overall eIF4E levels. This was not due to the inability of eIF4G to stabilize eIF4E, since if we co-expressed eIF4E and eIF4G, higher levels of eIF4E expression were observed (Figure 3A). Stabilization of overexpressed eIF4E depended on direct binding of eIF4G to eIF4E since mutating the eIF4E binding site in eIF4G removed the ability of overexpressed eIF4G to stabilize the co-expressed eIF4E (Figure 3A).

One might expect that the increased levels of both eIF4G and eIF4E when they are co-expressed would lead to an even larger increase in cap-dependent translation than with eIF4G alone. However, this is not what was observed. Instead, the major effect of expressing eIF4E and eIF4G together was to inhibit the effect of eIF4G on the IRES, such that now the IRES/CAP ratio was slightly decreased (Figure 3B).

### The effect of eIF4G on translation is dependent on an upstream open reading frame

**Figure 4:**
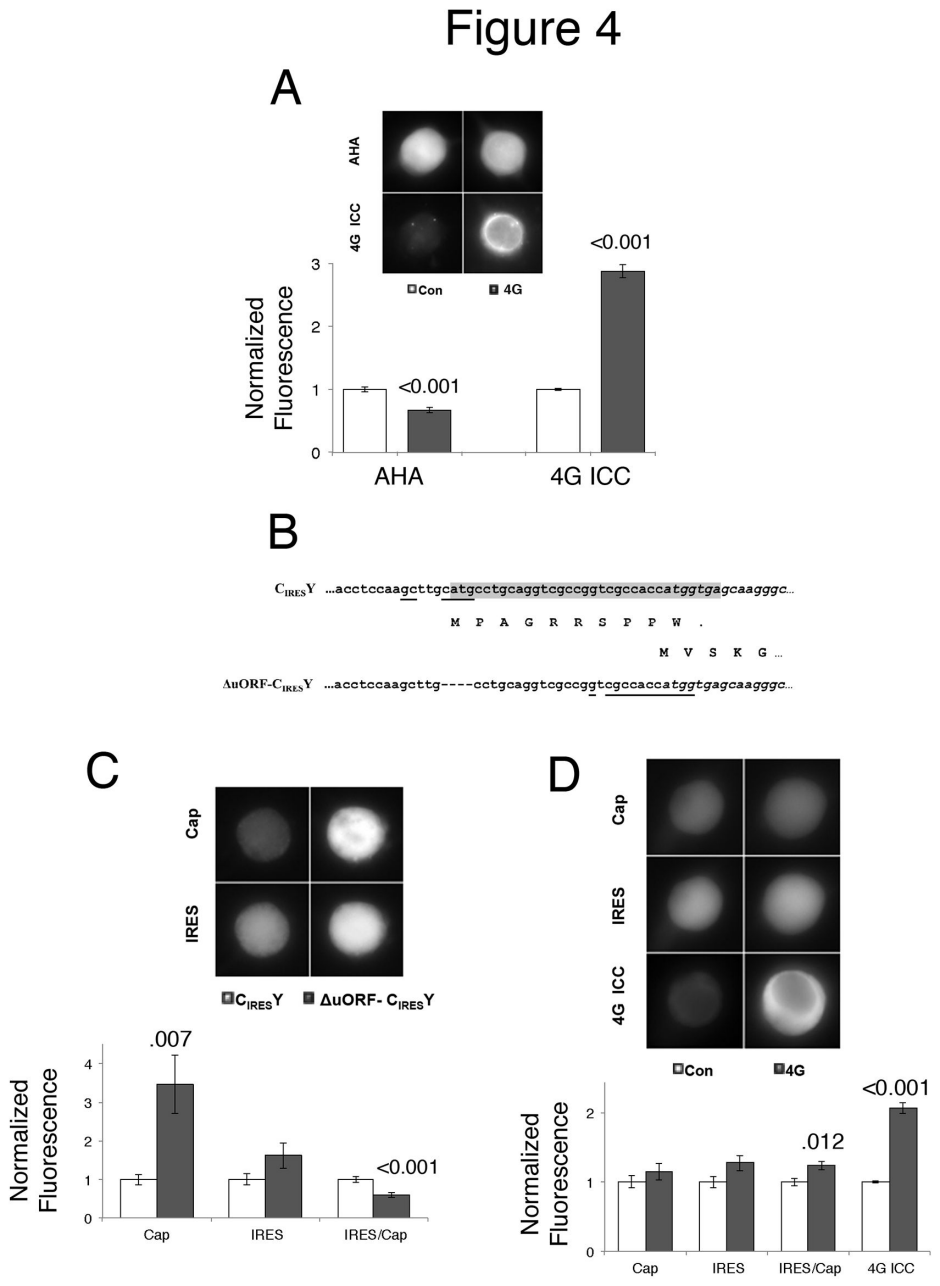
Overexpressed Ap4G only increases translation in the context of an upstream open reading frame (uORF). **A**) Cultured *Aplysia* sensory neurons were injected with either empty expression vector (Con) or Ap4G (4G). After 48 hours, overall translation was measured using AHA. In 3 of the 8 experiments, AHA labeling was done 12 hours after expression, but as these results were not significantly different than the results at 48 hrs, the results were combined into one group. Representative neurons show red fluorescence from incorporated AHA (AHA) and far red fluorescence from immunostaining for eIF4G (4G ICC) in the same neurons. Histogram is the means (normalized to Con) and SEMs from the groups of the representative neurons. The values, SEM and Ns are: Con (AHA 1.00 ± 0.04, n=98 neurons from eight experiments; 4G ICC 1.00 ± 0.02, n=102 neurons from eight experiments); eIF4G (AHA 0.67 ± 0.04, n=101 neurons from eight experiments, 4G ICC 2.87 ± 0.11, n-102 neurons from eight experiments.) P values from Student’s *t* test compared to control (with Welch’s and Bonferroni’s corrections) are shown over the bars. **B**) Deletion of uORF from bicistronic reporter construct. Beginning of eCFP ORF in italics, nucleotides that match the Kozak initiating consensus sequence are underlined and uORF is shaded. **C**) Cultured *Aplysia* sensory neurons were injected with either the original bicistronic construct (with uORF, C _IRES_Y) or the modified bicistronic construct (uORF deleted, ΔuORF- C _IRES_Y). Representative neurons (48 hours later) show cyan fluorescence (cap-dependent translation, Cap) and yellow fluorescence (IRES-dependent translation, IRES) in the same neurons. Histogram is the means (normalized to C _IRES_Y) and SEMs from the groups of the representative cells: The ratio of IRES- to cap-dependent translation was calculated (IRES/Cap). The values, SEM and Ns are: C _IRES_Y (Cap 1.00 ± 0.14, IRES 1.00 ± 0.15, IRES/CAP 1.00 ± 0.07, n=42 neurons from four experiments); ΔuORF- C _IRES_Y (Cap 3.47 ± 0.76, IRES 1.62 ± 0.33, IRES/CAP 0.60 ± 0.07, n=52 neurons from four experiments. P values from Student’s t-test compared to C _IRES_Y (with Welch’s and Bonferroni’s corrections) are shown over the bars. **D**) Cultured *Aplysia* sensory neurons were co-injected with the modified bicistronic fluorescent reporter (uORF deleted) and either empty expression vector (Con) or Ap4G (4G). Representative neurons (48 hours later) show cyan fluorescence (cap-dependent translation, Cap), yellow fluorescence (IRES-dependent translation, IRES) and red fluorescence from immunostaining for eIF4G (4G ICC) in the same neurons. Histogram is means (normalized to Con) and SEMs from the groups of the representative neurons. The ratio of IRES- to cap-dependent translation was calculated (IRES/Cap). The values, SEM and Ns are: Con (CAP 1.00 ± 0.09, IRES 1.00 ± 0.09, IRES/CAP 1.00 ± 0.04, 4G ICC 1.00 ± 0.01, n=122 neurons from twelve experiments); 4G (CAP 1.15 ± 0.12, IRES 1.28 ± 0.11, IRES/CAP 1.24 ± 0.06, 4GICC 2.07 ± 0.08, n=114 neurons from twelve experiments) P values from Student’s *t* test compared to control (with Welch’s and Bonferroni’s corrections) are shown over the bars.

To determine if overexpression of eIF4G generally increased translation, we used AHA, a methionine analog that can be coupled to fluorophores after fixation [12]. We first determined the conditions for stable, linear incorporation of AHA (Figure S2) and then measured the amount of translation after overexpression of eIF4G. Surprisingly, there was a small decrease in overall translation under these conditions (Figure 4A). This did not appear to be due to compensatory mechanisms, since even measuring translation soon after eIF4G expression did not reveal any increase in overall translation rates (data not shown). This suggests that endogenous eIF4G levels are saturating for overall translation and the decrease due to overexpression could be due to sequestering of other factors into unproductive complexes.

The results with overall translation conflicted with the results from the bicistronic vector suggesting a specific regulatory mechanism is present in this construct. We found an uORF in the 5’ UTR introduced by the polylinker (Figure 4B). This uORF of 10 amino acids ends after the initiating methionine of eCFP. Removing this uORF strongly increased cap-dependent translation from this construct (pNEX3∆uORF-C-_IRES_-Y) without significantly increasing IRES-dependent translation thereby significantly decreasing the IRES-CAP ratio (Figure 4C).

To determine if the effects of eIF4G on cap-dependent translation were due to the uORF, we determined the effect of overexpression of eIF4G on the translational reporter lacking the uORF (Figure 4D). Overexpression of eIF4G no longer increased cap-dependent translation in this construct. Surprisingly, there was also no significant effect of eIF4G on the level of IRES-dependent translation, although eIF4G still had a small, but significant effect on increasing the IRES/CAP ratio, similar to the ratio change in the presence of the uORF (Figure 2). It is possible that the lack of an increase with Ap4G expression is due to a ceiling on how much mRNA can be translated from this message. More fluorescent protein can be made though, by increasing the level of DNA injected (data not shown), suggesting that this is not a measurement ceiling.

**Figure 5:**
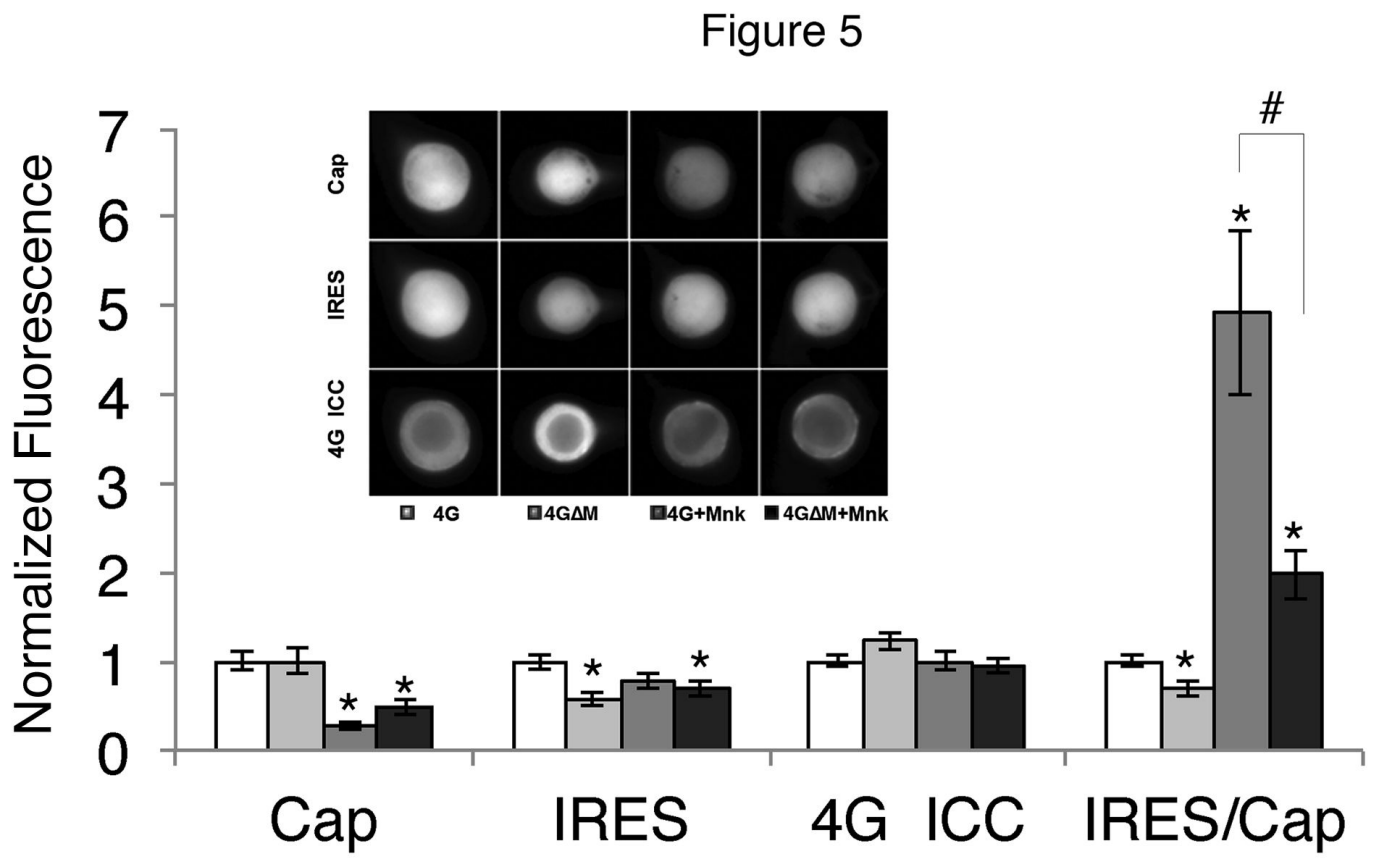
Mnk decreases cap-dependent translation independently of the uORF but required Ap4G binding. Cultured *Aplysia* sensory neurons were co-injected with the modified bicistronic fluorescent reporter (uORF deleted) and with either Ap4G (4G), Ap4G with mutated Mnk-binding site (4GΔM), Ap4G plus Mnk (4G+Mnk) or Ap4G with mutated Mnk-binding site plus Mnk (4GΔM+Mnk). Representative neurons (48 hours later) show cyan fluorescence (cap-dependent translation, Cap), yellow fluorescence (IRES-dependent translation, IRES) and red fluorescence from immunostaining for eIF4G (4G ICC) in the same neurons. Histogram is the means (normalized to 4G) and SEMs from the groups of the representative neurons:. In this experiment, the ratio of IRES- to cap-dependent translation was calculated (IRES/Cap). Values, SEMS and ns are: 4G (CAP 1.00 ± 0.10, IRES 1.00 ±0.09, 4G ICC 1.00 ± 0.07 IRES/CAP 1.00 ± 0.06, n= 53 neurons from three experiments); 4G∆M (CAP 1.01 ± 0.14, IRES 0.58 ± 0.08, 4G ICC 1.22 ± 0.09, IRES/CAP 0.68 ± 0.08, n=49 neurons from three experiments); 4G + Mnk (CAP 0.27 ± 0.05, IRES 0.77 ± 0.09, 4G ICC 1.00 ± 0.09. IRES/CAP 4.92 ± 0.93, n=39 neurons from three experiments) 4G∆M + Mnk (CAP 0.48 ± 0.08, IRES 0.69 ± 0.08, 4G ICC 0.95 ± 0.08, IRES/CAP 1.98 ± 0.27, n=53 neurons from three experiments). The groups were compared with a non-parametric Kuskal-Wallis ANOVA, followed by a post-hoc DUNN test to compare differences between all groups. CAP (KW statistic= 43.0, p<0.001, Both 4G + Mnk and 4G∆M + Mnk differ from control *, p<0.01) IRES (KW statistic = 13.4, p<0.01 Both 4G∆M and 4G∆M + Mnk differ from control *, p<0.05) 4GICC (KW statistic =6.2, p>0.05), IRES/CAP (KW statistic =94.8, p<0.0001, All groups differ from control * p<0.01, 4G∆M is different from 4G both in the presence and absence of Mnk, #, p<0.05).

We have earlier reported that one of the partners of eIF4G, the eIF4E kinase Mnk, when overexpressed could specifically inhibit cap-dependent translation and that this required the eIF4G binding site on Mnk. To explore this further, we compared the effect of overexpressed Mnk in the presence of eIF4G or a mutated eIF4G that could not bind Mnk (eIF4G∆Mnk). Due to the large number of groups, this experiment did not contain a group without eIF4G and the results are all normalized to overexpression of eIF4G. Mnk still strongly decreased cap-dependent translation using the pNEX3∆uORF-C-_IRES_-Y construct, suggesting that the effect of Mnk does not depend on the uORF (Figure 5). Moreover, the decrease in cap-dependent translation induced by Mnk overexpression was partially rescued by co-expression of eIF4G∆Mnk confirming that this effect of Mnk requires eIF4G binding. The incomplete rescue can be explained by the fact that endogenous eIF4G that can bind Mnk is still present in these experiments.

**Figure 6:**
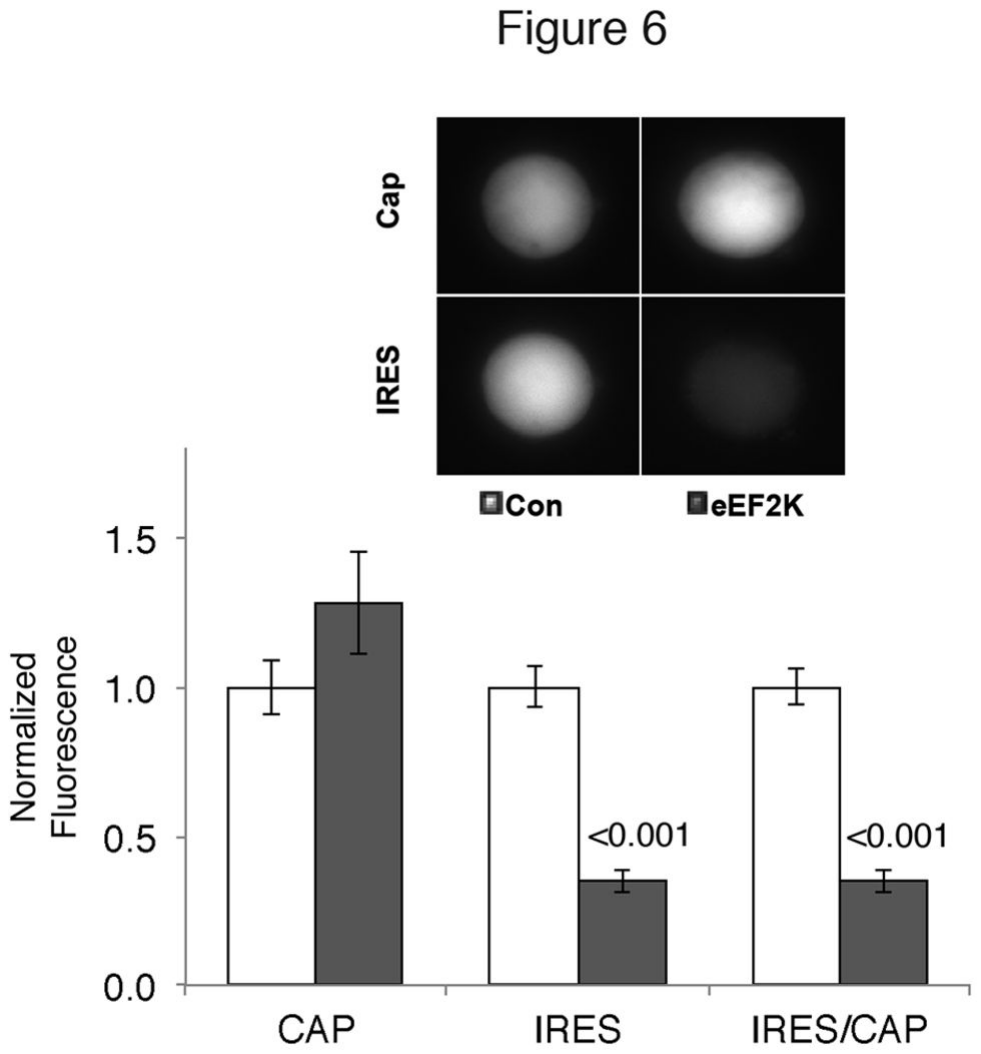
eEF2 kinase overexpression selectively inhibits IRES dependent translation independently of the uORF. Cultured *Aplysia* sensory neurons were injected with either empty expression vector (Con) or *Aplysia* eEF2K (eEF2K). Representative neurons (48 hours later) show cyan fluorescence (cap-dependent translation, Cap) and yellow fluorescence (IRES-dependent translation, IRES) in the same neurons. Histogram is the means (normalized to Con) and SEMs from the groups of the representative neurons. The ratio of IRES- to cap-dependent translation was calculated (IRES/Cap). Values, SEMS and ns are: Control (CAP 1.00 ± 0.09, IRES 1.00 ±0.07, IRES/CAP 1.00 ± 0.06, n =51 neurons from four experiments); eEF2K (CAP 1.28 ± 0.17, IRES 0.35 ± 0.02, IRES/CAP 0.35 ± 0.04, n=52 neurons from four experiments. P values from Student’s *t* test compared to control (with Welch’s and Bonferroni’s corrections) are shown over the bars.

Another earlier result with the bicistronic construct that could possibly be explained by the uORF involved the overexpression of eukaryotic elongation factor 2 kinase (eEF2K), which led to a small increase in cap-dependent translation coupled to a large decrease in IRES-dependent translation [17], despite the increased phosphorylation of eEF2 that should lead to a decrease in elongation of all messages. It has been proposed that this decrease in elongation could lead to an increase in specific transcripts that are rate-limited for initiation [5], and our results suggest that the uORF made translation of eCFP rate-limiting for eIF4G. However, eEF2 kinase had a similar effect on the bicistronic construct in the absence of the uORF (Figure 6), slightly increasing cap-dependent translation while decreasing IRES-dependent translation. The increase in cap-dependent translation was not significant in this experiment, while it was in the previous report [17] and this may be due to the increased basal translation of the reporter without the uORF. Nevertheless, the basic result that overexpression of eEF2K leads to a large change in the CAP/IRES ratio is still seen in the context of a reporter without the uORF.

## Discussion

A major goal of this research is to define the rate-limiting steps for translational control in the nervous system, using the *Aplysia* sensory neuron as a model system. While eIF4F production appears to be an important regulatory event, there is growing understanding that this regulation appears to be specific to some messages, as opposed to a mechanism for regulation of the overall translation rate. Here, we show that overexpression of eIF4G does not greatly increase translation of a reporter construct or of overall translation using incorporation of AHA. Together with our earlier results suggesting that eIF4E is not rate-limiting, we can conclude that overall translation rates in *Aplysia* sensory neuron do not appear to be limited by eIF4F levels.

In contrast, increases in eIF4G, but not eIF4E, were sufficient to increase translation in the context of an uORF. There are two possibilities for how translation of the eCFP reporter occurs in the presence of the uORF: reinitiation and/or leaky scanning. For reinitiation, the ribosome would resume scanning after a short uORF, presumably because the initiation factors are still complexed near to the site. However, this seems an unlikely explanation since in this case, the stop codon of the uORF is after the start codon of eCFP, and backward scanning is very inefficient [18]. In leaky scanning, the scanning ribosome bypasses an AUG, usually due to a suboptimal context for initiation. Indeed, the uORF AUG is not in an optimal Kozak consensus sequence (Figure 4B), increasing the probability for leaky scanning to occur [19]. Thus, we speculate that eIF4G increases translation of eCFP because of eIF4G’s ability to suppress initiation at the uORF’s suboptimal start site. This effect of eIF4G may be generalized, as has been shown previously for eIF4G in yeast in the context of the GCN4 promoter [20].

Interestingly, there have been a number of recent reports examining translation of ‘eIF4E’ sensitive mRNAs in the nervous system. This has been examined, either in the context of the loss of eIF4E-BP, overexpression of eIF4E, and use of the specific eIF4E-eIF4G inhibitor, eIF4GI. A number of specific mRNAs have been identified that are highly regulated by these conditions, including neuroligins, GluA1 and GluA2 [21,22]. All of these contain multiple uORFs and it is interesting to postulate that eIF4G-dependent suppression of these uORFs may contribute to the regulation of these mRNAs by eIF4F levels.

Another possibility is that overexpression of eIF4G generated incomplete eIF4F complexes and it was these complexes that led to increased read-through of the uORF, due to decreases in levels of 4G binding proteins associated with each eIF4F complex. The possibility that all eIF4F complexes do not contain stoichiometric amounts of each binding partner is supported by our work with Mnk. Increasing levels of Mnk greatly decrease cap-dependent translation, but this requires Mnk binding to eIF4G. If all eIF4F complexes normally contained Mnk, this would be difficult to explain.

How does the uORF in the bicistronic reporter affect previous results? We have reproduced the decrease in cap-dependent translation by Mnk and this did not depend on the uORF. Recently, we reported that overexpression of eEF2 kinase greatly decreased IRES-mediated translation and slightly increased cap-dependent translation, but this is also seen with the pNEX3∆uORF-C-_IRES_-Y construct. A number of studies have questioned the use of bicistronic reporters due to other explanations for translation of the second cistron independent of the IRES, such as cryptic promoters in the IRES, or splicing of the mRNA to generate a cap-dependent construct allowing expression of the IRES [23]. However, this is unlikely to be the case here: firstly, because sensitive tests for these alternative possibilities have been carried out [6] and secondly, because a large number of manipulations (increased levels of 4EBP, neuronal activity, eEF2 phosphorylation, Mnk overexpression) can dissociate cap-dependent translation from IRES dependent translation, a result that would not have been seen if both cistrons were translated in a cap-dependent manner [6–8,17]. While changes in mRNA levels cannot explain these differences since both cistrons are encoded by the same mRNA, it is conceivable that overexpression of eIF4G could increase both cap- and IRES-dependent translation through increasing levels of the mRNA encoding both cistrons. It is difficult to understand, however, how this could be removed by the deletion of the uORF. Unfortunately, we cannot measure levels of mRNAs accurately after injection of plasmid DNA in our single cell system.

One important remaining issue is how removing the uORF affected IRES-dependent translation. One possibility is that the presence of the uORF attracted initiation complexes to the mRNA that after termination of the short uORF could then be captured by the IRES in an eIF4G-dependent manner. Loss of the uORF reduced the number of these ‘unoccupied’ initiation complexes. Interestingly, the increase in IRES-dependent translation in the presence of the uORF did not occur when eIF4G and eIF4E were overexpressed. Overexpression of eIF4E in this context may have increased the rate of capture of these unoccupied initiation complexes by the cap. This model is consistent with the fact that removing the uORF did not significantly change translation of the IRES-dependent cistron (Figure 4C), as might be expected if the two cistrons were normally competing for rate-limiting factors.

## Supporting Information

Figure S1 **Characterization of *Aplysia* eIF4G antibody.** The C---terminal peptide sequence of *Aplysia* eIF4G (QLTQFFTWLSENEEPEAAS---COOH) was used to generate an antibody in rabbits. Homogenate proteins from fresh *Aplysia* ganglia were separated by PAGE (8%) and transferred to PVDF membrane which was incubated with antibodies purified from the serum of the *Aplysia* eIF4G---innoculated rabbits and visualized with ECL (Plus---ECL, Perkin---Elmer). Migration of protein markers are shown on left (kDa). An arrow points to the band predicted to be ApeIF4G at the predicted molecular weight of 195000 kDa.
(PDF)

Figure S2 **Characterization of AHA incorporation in *Aplysia* sensory neurons.** Cultured *Aplysia* sensory neurons were incubated in Met---reduced media (50 uM) for 120 minutes before adding AHA (50 uM) for 0 to 120 minutes. A paired dish of cells for each time point was incubated with emetine (250 uM) for 15 minutes before and during AHA incubation. Incorporated AHA was visualized by conjugating to an alkyne---fluorphore after fixing the cells. Representative neurons show red fluorescence from incorporated AHA at each time point. Graph shows mean net fluorescence at each time point; net fluorescence was calculated by subtracting the mean fluorescence from the group with emetine from the group without emetine at each time point. Graph shows representative experiment (>10 cells per point). Curve was fitted using a 3rd order polynomial equation with the origin as an endpoint.
(PDF)
