# Supplementary material for: Characterization of the Role of eIF4G in Stimulating Cap- and IRES-Dependent Translation in Aplysia Neurons
Source: PLoS One. 2013 Sep 3;8(9):e74085. doi: 10.1371/journal.pone.0074085 (PMC3760813; doi:10.1371/journal.pone.0074085)
Supplement: Figure S1 — Characterization of Aplysia eIF4G antibody. The C---terminal peptide sequence of Aplysia eIF4G (QLTQFFTWLSENEEPEAAS---COOH) was used to generate an antibody in rabbits. Homogenate proteins from fresh Aplysia ganglia were separated by PAGE (8%) and transferred to PVDF membrane which was incubated with antibodies purified from the serum of the Aplysia eIF4G---innoculated rabbits and visualized with ECL (Plus---ECL, Perkin---Elmer). Migration of protein markers are shown on left (kDa). An arrow points to the band predicted to be ApeIF4G at the predicted molecular weight of 195000 kDa. (PDF) [file pone.0074085.s001.pdf]

Figure S1

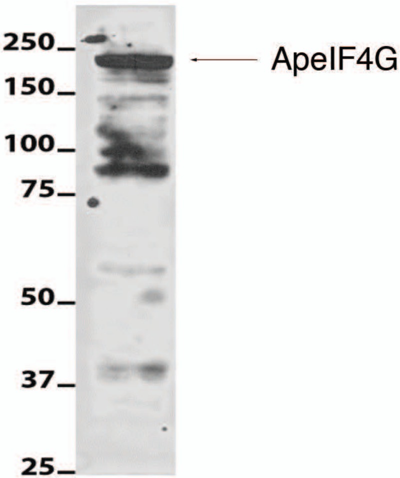

Fig. S1. Characterization of *Aplysia* eIF4G antibody. The C-terminal peptide sequence of *Aplysia* eIF4G (QLTQFFTWLSENEEPEAAS-COOH) was used to generate an antibody in rabbits. Homogenate proteins from fresh *Aplysia* ganglia were separated by PAGE (8%)and transferred to PVDF membrane which was incubated with antibodies purified from the serum of the *Aplysia* eIF4G-innoculated rabbits and visualized with ECL (Plus-ECL, Perkin-Elmer). Migration of protein markers are shown on left (kDa). An arrow points to the band predicted to be ApeIF4G at the predicted molecular weight of 195000 kDa.
