# Supplementary material for: Characterization of the Role of eIF4G in Stimulating Cap- and IRES-Dependent Translation in Aplysia Neurons
Source: PLoS One. 2013 Sep 3;8(9):e74085. doi: 10.1371/journal.pone.0074085 (PMC3760813; doi:10.1371/journal.pone.0074085)
Supplement: Figure S2 — Characterization of AHA incorporation in Aplysia sensory neurons. Cultured Aplysia sensory neurons were incubated in Met---reduced media (50 uM) for 120 minutes before adding AHA (50 uM) for 0 to 120 minutes. A paired dish of cells for each time point was incubated with emetine (250 uM) for 15 minutes before and during AHA incubation. Incorporated AHA was visualized by conjugating to an alkyne---fluorphore after fixing the cells. Representative neurons show red fluorescence from incorporated AHA at each time point. Graph shows mean net fluorescence at each time point; net fluorescence was calculated by subtracting the mean fluorescence from the group with emetine from the group without emetine at each time point. Graph shows representative experiment (>10 cells per point). Curve was fitted using a 3rd order polynomial equation with the origin as an endpoint. (PDF) [file pone.0074085.s002.pdf]

Figure S2

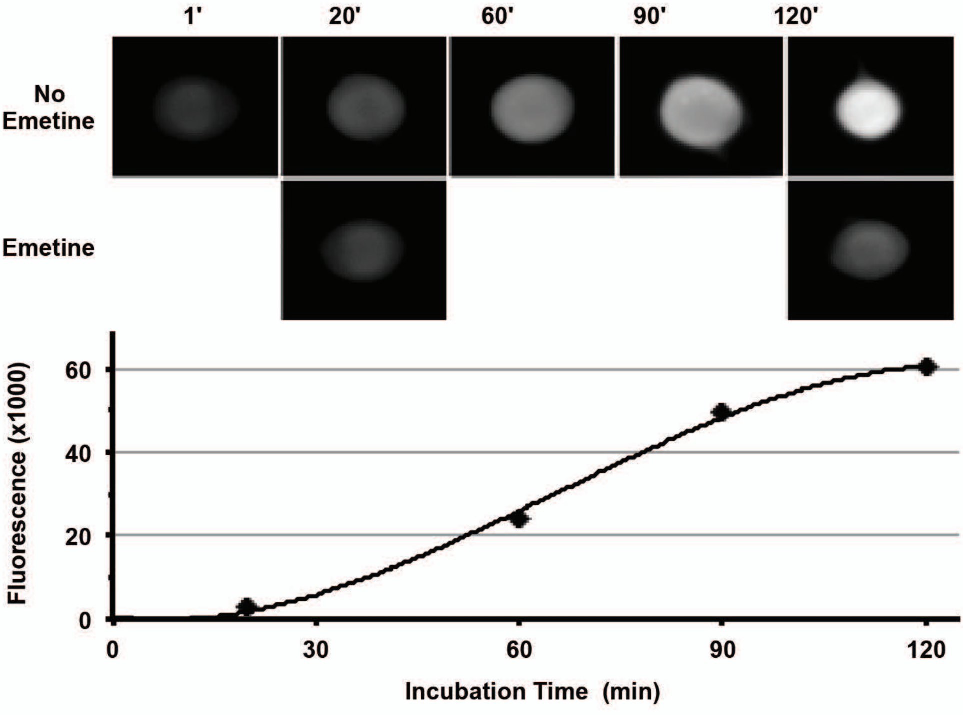
1'
20'
60'
90'
120'
No Emetine
Emetine
Fluorescence (x1000)
60
40
20
0
0
30
60
90
120
Incubation Time (min)

Fig. S2. Characterization of AHA incorporation in *Aplysia* sensory neurons. Cultured *Aplysia* sensory neurons were incubated in Met-reduced media (50 uM) for 120 minutes before adding AHA (50 uM) for 0 to 120 minutes. A paired dish of cells for each time point was incubated with emetine (250 uM) for 15 minutes before and during AHA incubation. Incorporated AHA was visualized by conjugating to an alkyne-fluorphore after fixing the cells. Representative neurons show red fluorescence from incorporated AHA at each time point. Graph shows mean net fluorescence at each time point; net fluorescence was calculated by subtracting the mean fluorescence from the group with emetine from the group without emetine at each time point. Graph shows representative experiment ($\geq$10 cells per point). Curve was fitted using a 3rd order polynomial equation with the origin as an endpoint.
